# Music Alters Visual Perception

**DOI:** 10.1371/journal.pone.0018861

**Published:** 2011-04-21

**Authors:** Jacob Jolij, Maaike Meurs

**Affiliations:** Vision and Cognition Group, Department of Experimental Psychology, Faculty of Behavioural and Social Sciences, University of Groningen, Groningen, The Netherlands; City of Hope National Medical Center and Beckman Research Institute, United States of America

## Abstract

**Background:**

Visual perception is not a passive process: in order to efficiently process visual input, the brain actively uses previous knowledge (e.g., memory) and expectations about what the world should look like. However, perception is not only influenced by previous knowledge. Especially the perception of emotional stimuli is influenced by the emotional state of the observer. In other words, how we perceive the world does not only depend on what we know of the world, but also by how we feel. In this study, we further investigated the relation between mood and perception.

**Methods and Findings:**

We let observers do a difficult stimulus detection task, in which they had to detect schematic happy and sad faces embedded in noise. Mood was manipulated by means of music. We found that observers were more accurate in detecting faces congruent with their mood, corroborating earlier research. However, in trials in which no actual face was presented, observers made a significant number of false alarms. The content of these false alarms, or illusory percepts, was strongly influenced by the observers' mood.

**Conclusions:**

As illusory percepts are believed to reflect the content of internal representations that are employed by the brain during top-down processing of visual input, we conclude that top-down modulation of visual processing is not purely predictive in nature: mood, in this case manipulated by music, may also directly alter the way we perceive the world.

## Introduction

One of the key tasks of the visual system is to provide the brain's executive systems with a stable, meaningful representation of the visual environment. This is not a trivial task, though: sensory input is often noisy, and more often than not open to several interpretations. In order to effectively process visual input, top-down information processing plays an important role in visual perception. The brain actively uses contextual information to generate a priori hypotheses about the most likely interpretation of specific sensory input [1]–[5]. For example, if you are in a meadow, a black-and-white pattern hitting your retina quite likely signals presence of a cow. According to a number of recent neuroimaging studies, the context of a meadow would pre-activate higher-level representations of object commonly encountered in meadows, such as cows. This information is fed back to earlier visual areas and compared with the actual perceptual input [2]–[5].

Top-down effects do not only speed up perceptual processing, they also affect perceptual awareness. In a recent psychophysical investigation, it was shown that observers perceive the color of a black-and-white picture of a banana as slightly yellowish – an effect attributed to a top-down filling in process, guided by memory: you know that bananas are yellow, so the brain fills in that color automatically [6]. Indeed, the notion of a ‘Bayesian brain’ – the idea that the brain is continuously predicting the most likely interpretation of new visual input on basis of context and memory, providing priors for analysis – gained a large amount of support in the recent literature [4].

However, the ‘Bayesian priors’ used in visual information processing are not set by perceptual memory alone. Higher level concepts, such as expectancy can affect subjective perception, too. When observers are led to believe that wearing a pair of glasses that have no effect on perception will make them see a multistable stimulus in one specific way, they overwhelmingly report that specific percept [7]. Moreover, in challenging stimulus detection tasks observers sometimes strongly believe to have seen stimuli that in fact have not been presented at all. Such misperceptions are also attributed to top-down processes [8]–[10].

Apparently, how we perceive our environment is for a large part determined by what we think. However, perceptual processing is not only influenced by cognitive or meta-cognitive processes. Emotion, and in particular mood, have been demonstrated to have an equally strong effect on perceptual processing. Mood affects the focus of processing: negative mood is associated with ‘local’ processing, whereas positive mood is associated with ‘global’ processing. When participants have to retell a brief story they had to memorize, participants in a negative mood tend to report details, whereas participants in a positive mood tend to report the gist of the story. Interestingly, in perceptual processing, a similar effect is observed. In an experiment in which participants had to rate whether a target object was more similar to an object that matched its global features or an object that matched its local features, participants in a negative mood showed a bias for the local features, whereas participants in a positive mood showed a bias for global features [11].

Not surprisingly, mood also has profound effects on the processing of emotional visual stimuli. Humans show a bias towards negative emotional material, the so-called negativity bias. This bias is already evident within the earliest stages of perceptual processing of pictures with emotional content. A positive mood however, induced by a brief music clip played right after presentation of an emotional picture, overcomes this negativity bias, as shown using brain evoked potentials [12]. Mood also affects the processing of facial expressions: when a face slowly morphs from a positive to a negative emotional expression, participants in a negative mood will indicate that the facial expression becomes negative at an earlier moment than participants in a positive mood [13], [14]. In ambiguous schematic facial expressions, mood, as induced with music, affects classification: whereas participants in a positive mood are more likely to judge the expression of an ambiguous face as positive, participants in a negative mood will judge that same face to have a negative emotional expression [15], [16]. These effects are not limited to facial expressions. A recent study found similar effects for emotional body language: participants were more accurate in identifying emotional body language that was congruent with their mood than incongruent emotional body language [17].

Summarizing, our visual representation of the world around us is not only affected by what we think or what we believe. How we feel has a profound impact on the ‘picture in our mind’ as well. In the present study, we further investigate the effect of mood, as induced by music, on top-down processing of visual information. We let observers do a difficult stimulus detection task, in which they had to detect schematic emotional faces embedded in noise, whilst listening to music that made them happy or sad. Critically, we left out the targets in half of all trials. In line with earlier studies, we found strong mood-congruency effects, that is, participants were better in detecting mood-congruent faces. However, participants also made a significant number of false alarms. The content of these illusory percepts was also strongly modulated by mood, showing that mood not only enhances sensitivity to mood-congruent features in visual input, but can even determine content of visual perception in absence of real visual input.

## Materials and Methods

### Ethics statement

The study was approved by the local Ethics Committee (“Ethische Commissie van het Heymans Instituut voor Psychologisch Onderzoek”) and conducted according to the Declaration of Helsinki. Written informed consent was obtained from all observers.

### Observers

Observers were 43 healthy first year students (17 males, mean age 20.7 years, SD 1.2 years) in the International Bachelor's Programme in Psychology of the University of Groningen with normal or corrected-to-normal vision.

### Visual stimulation

Stimuli were constructed using Matlab R14 (The Mathworks Inc., Natick, Massachusetts), and consisted of a 140×140 pixels array of random noise, with a circle with a diameter of 74 pixels present in the centre, with slightly elevated contrast. All 255 values of the grayscale palette were used to construct the random noise images. Target stimuli contained also a pair of eyes, eyebrows, and a mouth within the circle, forming a schematic face with a sad or a happy expression. Schematic faces have been shown to carry similar emotional content as real faces, and may thus be used to study the effects of emotion in perception tasks [18].

Stimulus presentation was done using in-house written software (Taskmania, University of Groningen, The Netherlands). Stimuli were presented on a 19″ Philips Brilliance 190B TFT monitor (Philips BV, Eindhoven, The Netherlands) with a resolution of 800×600 pixels; viewing distance was approximately 80 cm. Trials were animations of 9 frames, with each frame presented for approximately 107 ms. The fifth frame of each animation was accompanied by a cue (a grey square around the noise patch) and contained a target stimulus in 50% of all trials. Target presence and target identity (happy or sad) were chosen at random in each trial to avoid expectancy or learning effects. Noise patches were chosen at random from a series of 500 different noise images; targets, if present, were chosen from a series of 50 happy and 50 sad images. Please note that only the noise pattern added to these images was different – the schematic faces were identical in all trials in which a real target was presented.

After each trial, participants had to indicate whether they had seen a sad face or a happy face by using the computer keyboard (the P and Q keys, respectively), or to withhold from responding when they had not seen a face. They were specifically instructed to be conservative with their responses, i.e. to only respond when they were absolutely sure to have seen a face. No feedback was given.

### Music manipulation

Participants were instructed to bring at least 15 minutes of songs that made them feel happy and 15 minutes of songs that made them feel sad. They were left completely free in their choice of musical style, and indeed the material widely varied between subjects (e.g. ‘Under the Bridge’ by The Red Hot Chili Peppers or Mozart's ‘Requiem’).

### Mood assessment

Participants' mood was assessed using the Self Assessment Manakin, a non-verbal assessment technique to measure the pleasure, arousal, and dominance associated with a person's affective state, and is widely used in emotion research [19]. We have specifically chosen a non-verbal assessment tool: participants were recruited from an International Bachelor's Programme, and native languages of participants differed. Using a non-verbal tool avoids problems due to translation or interpretation as result of differences in fluency in English.

### Experimental procedure

The experiment consisted of three experimental conditions: a condition in which the participants performed the task without music (no music condition), a condition in which they listened to happy music, and a condition in which they listened to sad music. Participants listened to their own material whilst performing two blocks of 100 trials of the task in each of the three conditions (no music, sad music, happy music). Each condition lasted at most 10 minutes, and music was played throughout the entire condition. Music was played via an MP3-player over headphones. Participants chose a volume that they found comfortable. The order of the conditions was counterbalanced over all participants. Participants' mood was assessed using the SAM at the start of the experiment, and immediately after each condition.

### Analyses

Data were analyzed using SPSS version 16.0 (SPSS Inc., Natick, USA). Repeated measures ANOVAs, with type of music as within-subject factor with three levels (no music, happy music, sad music), were conducted on the three scales of the SAM (valence, dominance, and arousal), the detection and identification rates for happy and sad faces, the proportion of false alarms, and the proportion of false alarms classified as happy faces.

## Results

Music significantly affected mood, as measured with the valence scale of the SAM, F(2, 36) = 43,639, p = 0.00000. Participants reported a significantly more positive mood as compared with the no music condition after listening to happy music (p = 0.0000), and a more negative mood after listening to sad music (p = 0.00065). Arousal was also significantly elevated by music, F(2, 36) = 3,26, p = .044; there was no significant difference between happy and sad music. No significant effects on the dominance scale of the SAM were observed.

**Figure 1:**
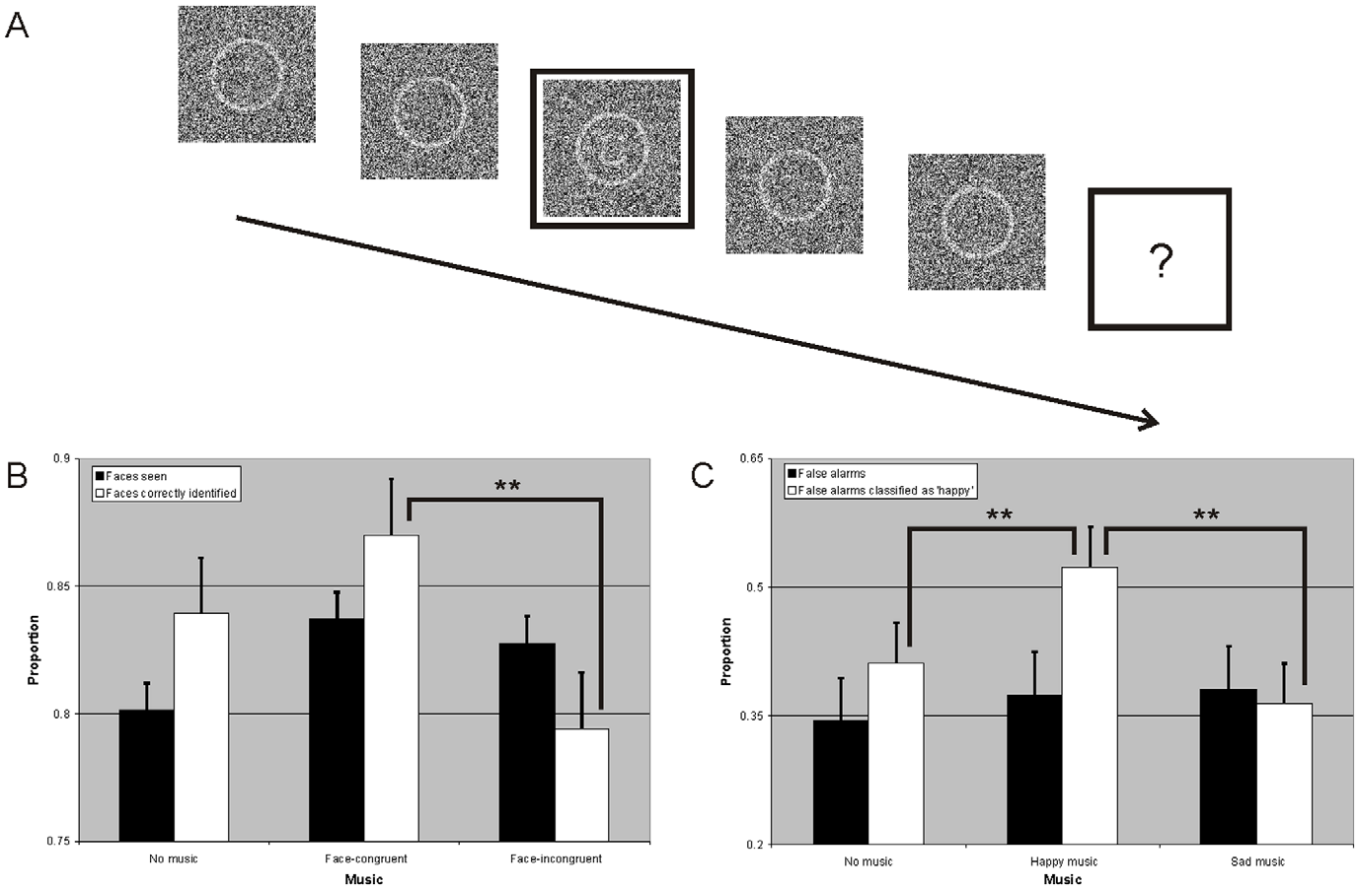
Experimental design and results. **a**. Schematic of a single trial. Patches of noise were presented for 107 ms, creating an animation of dynamic noise; the middle patch contained either a face stimulus or just noise and was accompanied by an annulus surrounding the patch. After the trial, observers had to indicate whether they had seen a happy, a sad or no face. **b**. Proportions of real faces seen (black bars) and correctly identified emotional expressions (white bars). Error bars indicate 1 S.E. (n = 42), asterisks (**) indicate significant difference at p<.001. **c**. Proportions of reported false alarms (black bars) and proportion false alarms classified as happy (white bars). Error bars indicate 1 S.E. (n = 42), asterisks (**) indicate significant difference at p<.001.

Overall, participants performed the stimulus detection task very well (average correct detection and identification rate were both 83%). Stimulus detection rates were elevated in the music conditions as compared with the no music condition, albeit not significantly (no music 80%, music-congruent faces 84%, music-incongruent faces 83%; F(2, 36) = 2.817; p = 0.065). Moreover, music did have a significant effect on emotion identification (F(2, 36) = 10.200; p = 0.00000): while listening to happy music, participants were more accurate in detecting happy faces then when listening to sad music and vice versa (87% vs. 79%; p = 0.00029) – see figure 1b. This is consistent with earlier reported effects of mood congruency in emotion detection [13]–[17].

However, we also found strong effects of music in trials in which no real stimulus was presented. Participants reported slightly more false alarms in the music conditions than in the no music condition, but this effect was not significant (no music 34%, happy music 38%, sad music 38%; F(2, 36) = 1,357; p = 0.263). However, whether people reported a happy face or sad face while in fact there was none was strongly affected by music: the proportion of happy faces reported was significantly higher in the happy music condition than in the other two conditions (no music 40%, happy music 53%, sad music 36%; F(2, 36) = 9.969, p = 0.00036) – see figure 1c. An additional analysis of all noise images in which observers reported to have seen a face revealed no evidence of any randomly present structure in the noise images, suggesting that perception of faces in this experiment was mainly based on internally generated signals, and not on randomly present features that appeared like a face in the noise stimuli [8].

## Discussion

We have shown that in a challenging stimulus detection task, participants' conscious reports about stimuli are not only affected by previous knowledge and expectation, but also by mood. Participants were more sensitive to mood-congruent emotional stimuli, and showed a bias towards reporting false alarms that were mood-congruent in trials in which no stimuli were presented. These results are in line with the earlier discussed studies on the effects of mood on visual processing: observers become more sensitive to facial features that are congruent with their own emotional state [13]–[17]. However, we extend this earlier work by demonstrating that this bias extends to illusory perception: even when there is no real stimulus present, mood affects what participants see.

Mood congruency effects in emotion perception may be explained in two different ways: first, it may be the case that post-perceptual decision processes are biased. In that case, the perceptual representation of an emotional face is not affected by mood – only post-perceptual processing is, for example, by an adjustment of decision thresholds [cf. 20]. However, it may also be that mood affects perceptual processing itself, similar to context-dependent top-down modulation of visual processing [1]–[5]. In such an interpretation, the sensory representations on which a subsequent perceptual decision would be based would be directly influenced by the observer's state of mind.

Our data seems to favour this latter hypothesis. Apart from replicating earlier work on increased sensitivity to mood-congruent facial expressions, we report that observers' reports during false alarms were also strongly biased by mood. Given that our participants were instructed to only report presence of a face when they were absolutely sure a face was presented, it is reasonable to assume these false alarms were truly reflect ‘illusory percepts’, as opposed to guesses in response to an ambiguous stimulus. Several studies have attributed illusory percepts to ‘pure top-down’ processing, and indeed report activity in visual areas when participants report to have seen a highly expected stimulus while in fact no stimulus has been presented [8]–[10], [21].

Extrapolating this to the present results, we may assume that top-down modulation of perceptual processing is not just driven by visual context, memory, or expectation. Apparently, emotional state can also influence processing of perceptual input in a top-down manner, and alter the contents of visual awareness, even in absence of structured visual input. Mood dependency of top-down processing would go against the idea that top-down modulations are purely ‘predictive’ or ‘Bayesian’ in nature: the mood of the observer has obviously no effect on the likelihood of encountering mood-congruent emotional stimuli. So, what may be the functional significance of mood-dependent top-down modulation of visual processing?

It is interesting to note that recent studies have found modulation of early visual cortex activity as a function of subjective reward value [22]. It is not unlikely that our findings represent a similar mechanism, but now for emotional facial expressions – the ‘reward value’ an observer attaches to an emotional face obviously changes as functions of that observer's own emotional state. Possibly, such a mechanism aids in fine-tuning the interpretations of others' emotions, respective to our own. A recent brain imaging study has shown that congruency between one's own mood, as induced by music, and a presented emotional face results in a specific pattern of brain activation: congruency activates the superior temporal gyrus, but diminishes activity in the face-specific fusiform gyrus, whereas the opposite patterns is observed for incongruent music-face pairs [16]. This seems to suggest a difference in top-down control in visual face processing when the observer's mood is incongruent with the emotional expression of target faces as opposed for mood-congruent faces. Moreover, disorders in top-down processing of faces have been linked to autism spectrum disorder, suggesting an important role for top-down processing of socially relevant stimuli, such as emotional faces, in social cognition [23]. Together with the present results, these findings suggest that the image in our mind may in part be determined by social cognitive processes, although the exact underlying mechanisms remain unclear.

### Conclusion

Our results show that our conscious experience of the world may be less objective than we think. Conscious experience does not only reflect ‘what is out there’, but also our previous knowledge and expectation [1]–[10], [21]. Our findings show that mood, as induced by music, is also reflected in visual awareness, both in biasing processing sensory input, as in the generation of conscious visual percepts in absence of structured visual input. In other words, the music you are listening to might directly alter the way you perceive the world.
